# Non-HLA antibody trajectories may predict kidney transplant outcomes

**DOI:** 10.3389/fimmu.2026.1729129

**Published:** 2026-02-24

**Authors:** Raja Rajalingam, Tara K. Sigdel, Stalinraja Maruthamuthu, Pawan Kumar Raghav, Zoltan G. Laszik, Gilberto Da Gente, Denice Kong, Jun Shoji, Bryan Ray

**Affiliations:** 1 Immunogenetics and Transplantation Laboratory, Department of Surgery, University of California, San Francisco, CA, United States; 2 Department of Surgery, University of California, San Francisco, CA, United States; 3 Department of Pathology, University of California, San Francisco, CA, United States; 4 Cedars-Sinai Medical Center, Los Angeles, CA, United States; 5 Research and Development, Werfen, Wakesha, WI, United States

**Keywords:** alloimmunity, antibody-mediated rejection (ABMR), donor-specific antibodies (DSA), graft outcomes, immune monitoring, kidney transplantation, minor histocompatibility antigens, non-HLA antibodies

## Abstract

**Introduction:**

Donor-specific HLA antibodies (DSAs) are well-established mediators of antibody-mediated rejection (AMR), but growing evidence suggests that non-HLA antibodies may also contribute to kidney allograft dysfunction. However, the clinical utility of non-HLA antibody testing remains limited due to assay variability and undefined pathogenic thresholds.

**Methods:**

In this longitudinal study, we evaluated 167 deceased-donor kidney transplant recipients at UCSF using a multiplex Luminex assay to quantify IgG reactivity against 88 non-HLA antigens in paired pre- and post-transplant serum samples (n = 334). Patients were stratified by HLA-DSA status and graft outcome. The non-HLA panel reactive antibody (PRA) percentage was defined as the proportion of positive beads among the 88 tested antigens.

**Results:**

Global non-HLA PRA declined significantly from pre- to post-transplant (9% to 6%; *P* = 0.0010) only among recipients with functioning grafts, driven by reduced reactivity to signaling, cytoskeletal, and immune-related antigens. Elevated pre-transplant non-HLA PRA was associated with prior sensitization, IgA nephropathy, and African American ancestry, but was independent of age, ABO type, and HLA cPRA. AMR occurred primarily in recipients with both preformed and *de novo* DSA, while no rejection was observed in DSA-negative recipients. Death-censored graft survival varied across groups, and neither preformed nor *de novo* DSA consistently predicted outcomes.

**Discussion:**

These findings suggest that longitudinal trajectories of non-HLA PRA, rather than staticmeasurements or DSA status alone, may influence graft loss. The dynamic nature of non-HLA antibody responses and supports integration of non-HLA antibody profiling into longitudinal immune monitoring frameworks. Larger multicenter studies are needed to define clinically meaningful threshold and estabilish the utility of non-HLA PRA testing across diverse transplant populations.

## 1 Introduction

Human leukocyte antigens (HLA) have long been central to transplant immunology, serving as key determinants of graft survival and predictors of antibody-mediated rejection (AMR) in both solid organ and hematopoietic stem cell transplantation (1–4). Donor-specific HLA antibodies (DSAs) are established mediators of AMR; however, growing evidence indicates that antibodies directed against non-HLA antigens also play a significant role in graft injury, particularly in kidney, heart, and lung transplantation (5–13). Their detection may provide critical insights into graft pathology, improve risk stratification before and after transplantation, facilitate earlier identification of immune activation, and inform individualized immunosuppressive strategies (14–16). Despite their potential, non-HLA antibodies are not currently included in routine transplant assessment or allocation protocols. While their role in DSA-negative rejection is increasingly acknowledged, the lack of validated assays and clinically actionable biomarkers continues to limit their broader clinical application.

Reflecting this evolving understanding, the Banff 2017 update expanded the definition of AMR to include tissue injury associated with both HLA and non-HLA antibodies (17). Non-HLA antibodies encompass a heterogeneous group of allo- and autoantibodies directed against antigens such as MICA, angiotensin II type 1 receptor (AT1R), endothelin receptor type A (ETAR), vimentin, perlecan, and endothelial cell components. Despite their potential clinical relevance, non-HLA antibody testing remains largely limited to research settings, constrained by the lack of standardized assays and incomplete knowledge regarding their pathogenic thresholds and clinical significance (18–21). Moreover, while endothelial cells are frequently considered principal targets of non-HLA reactivity, endothelial crossmatch assays have shown limited correlation with rejection outcomes in kidney transplantation (22).

Recent advances in multiplexed Luminex single antigen bead-based technology offer new opportunities for broader and more systematic detection of non-HLA antibodies. Among these, a commercially available panel of 60 unique non-HLA antigens has demonstrated promise for comprehensive profiling. However, its utility in routine clinical practice and its predictive value for transplant outcomes remain to be fully established.

Here, we employed this multiplexed Luminex assay to longitudinally evaluate non-HLA antibody profiles in deceased donor kidney transplant recipients. The aims of this study were to (i) investigate potential triggers of their development, including pregnancy and transplantation, (ii) determine whether non-HLA antibodies are pre-existing or develop *de novo* after transplantation, (iii) evaluate their relationship with crossmatch reactivity using blood vessel–derived endothelial cells, and (iv) assess their association with kidney allograft outcomes.

We hypothesized that the temporal dynamics—or trajectory—of non-HLA antibodies, rather than their presence alone, carry meaningful prognostic information not captured by conventional HLA metrics. Our findings suggest that non-HLA alloimmunity follows an independent and clinically actionable pathway, and that dynamic non-HLA antibody profiling may enhance post-transplant risk assessment and inform early immunosuppressive interventions.

## 2 Materials and methods

### 2.1 Study design and patient cohort

This retrospective, observational cohort study analyzed biobanked serum samples from deceased donor kidney transplant recipients at the University of California, San Francisco (UCSF). The study was conducted in accordance with the Declaration of Helsinki and approved by the UCSF Institutional Review Board (IRB #16-19103). Due to its retrospective nature, patients were not randomized but were grouped according to clinically relevant immunologic characteristics that reflect real-world transplant conditions.

**Figure 1:**
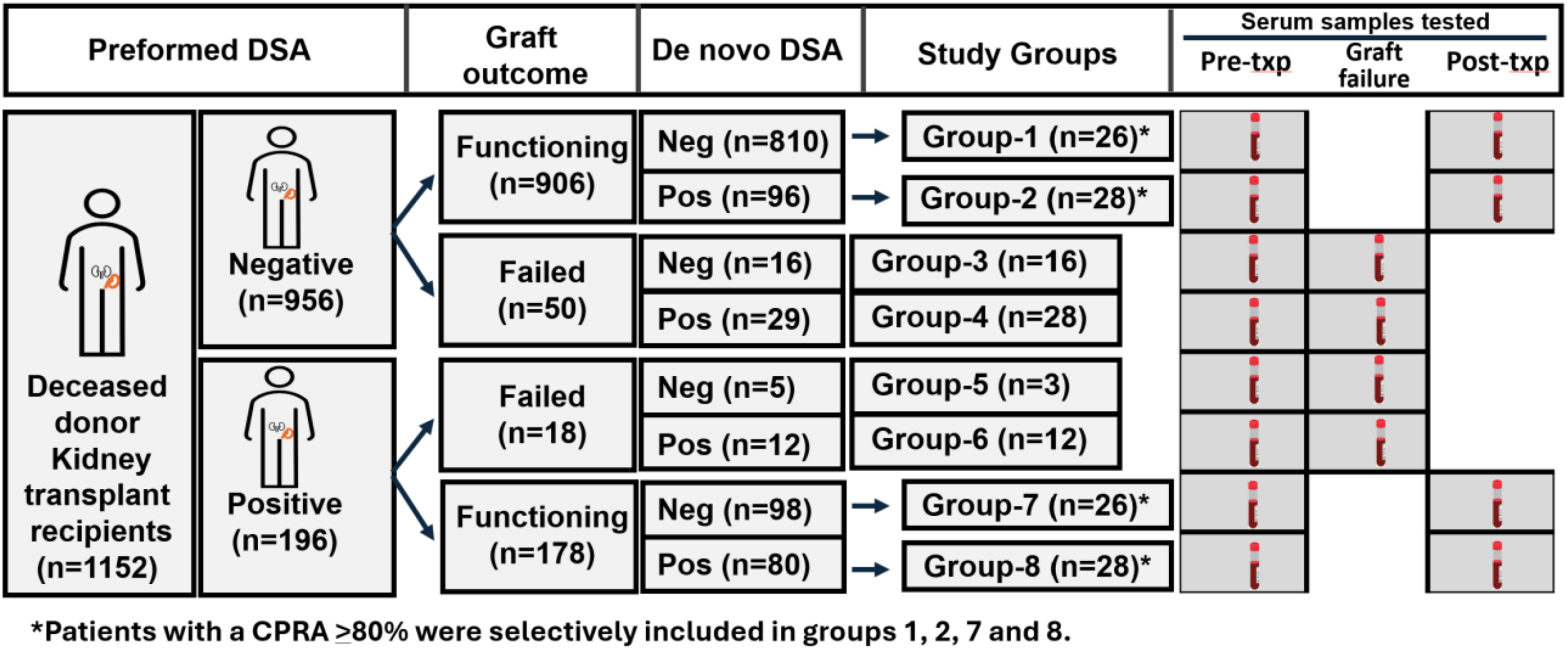
Study cohort. A total of 167 kidney transplant recipients, representing eight distinct study groups, were selectively included based on the presence of preformed donor-specific antibodies (DSA), development of *de novo* DSA, and graft outcomes. Blood samples were collected within 3 months prior to transplantation and at 6 months post-transplant, and were analyzed for non-HLA antibodies using a single non-HLA antigen bead assay. Demographic and clinical characteristics of each study group are summarized in **Table 1**.

A total of 1152 consecutive patients who received a deceased donor kidney transplant between January 2013 and December 2018 were screened (**Figure 1**). Patients younger than 18 years or those undergoing multi-organ transplants involving a kidney were excluded. Based on the presence or absence of preformed donor-specific HLA antibodies (pfDSAs), patients were categorized into two groups: pfDSA-negative (n = 956) and pfDSA-positive (n = 196). This categorization captures established pre-transplant immunologic risk. Each group was further stratified by graft outcome (functioning vs. failed) to evaluate how antibody profiles correlated with graft performance and subsequently subdivided based on the presence or absence of *de novo* donor-specific antibodies (dnDSA) to assess the contribution of post-transplant sensitization. This grouping strategy was designed to maximize scientific relevance and interpretability within the practical constraints of cost and biobanked sample availability, providing a clinically grounded, hypothesis-generating framework for investigating non-HLA antibody kinetics and immune dynamics in kidney transplantation.

Among the 68 patients with graft failure, 59 with both pre- and post-transplant serum samples available for non-HLA antibody testing were included in this study. From recipients with functioning grafts, highly sensitized individuals with a calculated panel reactive antibody (cPRA) ≥ 80% were identified, and 108 patients were selected—balanced between those with and without dnDSA (n = 26 or 28 per group)—to improve comparability in baseline immunologic risk across groups. This selection resulted in a final cohort of 167 patients representing eight distinct groups (Groups 1–8; **Figure 1**). A total of 334 serum samples—collected within 3 months pre-transplant and at 6 months post-transplant—were included for non-HLA antibody profiling. Demographic and clinical data are summarized in **Table 1**.

**Table 1:** Demographic and clinical characteristics of study groups stratified by preformed DSA, *de novo* DSA, and graft outcomes.

Recipient characteristics	All Patients	Group-1	Group-2	Group-3	Group-4	Group-5	Group-6	Group-7	Group-8
	(N=167)	(N=26)	(N=28)	(N=16)	(N=28)	(N= 3)	(N=12)	(N=26)	(N=28)
	Preformed DSA →	Negative	Negative	Negative	Negative	Positive	Positive	Positive	Positive
	*de novo* DSA →	Negative	Positive	Negative	Positive	Negative	Positive	Negative	Positive
	Graft survival →	Functioning	Functioning	Failed	Failed	Failed	Failed	Functioning	Functioning
	%	%	%	%	%	%	%	%	%
Age and sex									
#Age at transplant (years)	47 ± 114.3 [40, 59]	51 ± 13.5 [39, 59]	45 ± 16.0 [41, 61]	59 ± 13.5 [45, 66]	45 ± 15.0 [30, 56]	56 ± 4	42 ± 14 [39, 54]	53 ± 14 [44, 62]	43 ± 13 [40, 59]
Age at transplant: > 60 years	23.35	19.23	32.14	43.75	17.86	33.33	0.00	26.92	17.86
Female	64.07	69.23	78.57	43.75	50.00	100.00	66.67	57.69	71.43
Male	35.93	30.77	21.43	56.25	50.00	0.00	33.33	42.31	28.57
Race/ethnicity									
White	22.20	19.20	17.80	18.80	14.30	33.33	25.00	34.60	25.00
African-American	24.00	15.40	14.30	37.40	35.70	33.33	41.70	15.40	21.40
Hispanic/Latino	32.30	53.90	39.30	18.80	25.00	0.00	8.30	30.80	35.70
Asian	18.60	11.50	14.30	25.00	25.00	33.33	25.00	19.20	14.30
^a^Others	2.90	0.00	14.30	0.00	0.00	0.00	0.00	0.00	3.60
Blood type									
O	53.29	57.69	57.14	56.25	60.71	33.33	58.33	30.77	57.14
A	31.74	19.23	32.14	37.50	17.86	66.67	25.00	50.00	35.71
B	10.78	23.08	10.71	6.25	7.14	0.00	8.33	11.54	7.14
AB	4.19	0.00	0.00	0.00	14.29	0.00	8.33	7.69	0.00
^#^BMI at listing (kg/m2)	27 ± 5.8 [23, 31]	28 ± 4.2 [24, 30]	28 ± 6.3 [24, 33]	28 ± 5.6 [25, 35]	28 ± 6.3 [23, 31]	34 ± 9	28 ± 6.3 [23, 31]	27 ± 5.8 [22, 31]	26 ± 5.9 [22, 31]
<25 kg/m2	37.13	30.77	25.00	37.50	35.71	33.33	58.33	46.15	39.29
25-30 kg/m2	30.54	46.15	35.71	18.75	39.29	0.00	16.67	15.38	32.14
>30 kg/m2	32.34	23.08	39.29	43.75	25.00	66.67	25.00	38.46	28.57
Retransplants	41.32	46.15	14.29	12.50	25.00	100.00	58.33	57.69	67.86
^b^Pre-transplant HLA antibodies	0.00	0.00	0.00	0.00	0.00	0.00	0.00	0.00	0.00
<20% cPRA	7.19	0.00	0.00	31.25	17.86	33.33	8.33	0.00	0.00
20-79% cPRA	7.19	0.00	0.00	37.50	7.14	0.00	0.00	7.69	7.14
80-99% cPRA	39.52	42.31	57.14	12.50	39.29	0.00	16.67	53.85	35.71
100% cPRA	46.11	57.69	42.86	18.75	35.71	66.67	75.00	38.46	57.14
Highly sensitized: 80-100% cPRA	85.63	100.00	100.00	31.25	75.00	66.67	91.67	92.31	92.86
DSA in any pre-Txp samples	41.32	0.00	0.00	0.00	0.00	100.00	100.00	100.00	100.00
^#^Days on waitlist	1728 ± 1257 [839,2880]	1213 ± 1347 [617,2123]	1022 ± 1266 [190,2205]	2052 ± 1088 [1233,3026]	2127 ± 991 [1347,2911]	3144 ± 1497	1286 ± 1214 [847,2695]	2203 ± 1283 [928, 2883]	2107 ± 1345 [1053, 3243]
Pre-transplant dialysis									
On dialysis	86.83	92.31	78.57	81.25	92.86	100.00	83.33	76.92	96.43
#Days on dialysis	1694 ± 1984 [769, 2961]	1426 ± 1411 [981,1426]	1271 ± 1185 [227,2574]	2051 ± 1453 [1371,3290]	2809 ± 1591 [1147,3527]	3719 ± 1622	1342 ± 1409 [939,2924]	1374 ± 3626 [171,2958]	1812 ± 1762 [869,2845]
Dialysis <3 years	33.53	34.62	42.86	18.75	21.43	0.00	25.00	50.00	35.71
Dialysis >3 years	66.47	65.38	57.14	81.25	78.57	100.00	75.00	50.00	64.29
Diabetes at listing									
Type I	1.20	0.00	3.57	0.00	0.00	0.00	0.00	0.00	3.57
Type II	20.96	23.08	28.57	25.00	21.43	0.00	8.33	15.38	21.43
No diabetes	77.84	76.92	67.86	75.00	78.57	100.00	91.67	84.62	75.00
Diabetes status or type unknown	0.00	0.00	0.00	0.00	0.00	0.00	0.00	0.00	0.00
Primary diagnosis									
Diabetes Mellitus	16.17	19.23	28.57	18.75	21.43	0.00	8.33	7.69	7.14
Hypertensive Nephrosclerosis	20.96	11.54	28.57	37.50	25.00	33.33	16.67	11.54	17.86
Retransplant/Graft Failure	25.75	34.62	7.14	6.25	7.14	66.67	41.67	38.46	42.86
Polycystic Kidneys	5.39	0.00	7.14	18.75	3.57	0.00	8.33	0.00	7.14
Chronic Glomerulonephritis	7.19	7.69	0.00	0.00	10.71	0.00	8.33	11.54	10.71
IgA Nephropathy	2.99	7.69	10.71	0.00	0.00	0.00	0.00	0.00	0.00
Focal Glomerular Sclerosis	4.79	11.54	3.57	6.25	7.14	0.00	0.00	0.00	3.57
Systemic Lupus Erythematosus	2.40	0.00	3.57	0.00	7.14	0.00	0.00	3.85	0.00
Others	14.37	7.69	10.71	12.50	17.86	0.00	16.67	26.92	10.71

#Median+Standard Deviation (SD) [25%, 75% interquartile range].

^a^American Indians, Alaska Natives, Native Hawaiian, Pacific Islander and Multiracial. ^b^DQA1/DPA1/DPB1 antibodies are not currently considered for cPRA calculation.

To assess temporal changes in non-HLA PRA following kidney transplantation, a subset of 13 recipients was monitored longitudinally for 5–9 months with multiple post-transplant time points. This subgroup was selected based solely on the availability of serial post-transplant serum samples, independent of clinical outcomes or antibody status.

### 2.2 Immunosuppression protocol

Immunosuppressive therapy for deceased donor kidney transplantation in this cohort included induction with thymoglobulin (6 mg/kg; Genzyme, Cambridge, MA, USA). Recipients with preformed donor-specific antibodies (DSA) received an additional dose of intravenous immunoglobulin (IVIG, 1 g/kg) on postoperative days 1 and 2. Maintenance immunosuppression consisted of tacrolimus, mycophenolate mofetil, and prednisone.

### 2.3 Non-HLA antibody profiling

Non-HLA IgG antibodies were measured using a prototype Non-HLA Antibody bead set (Werfen,Waukesha, WI), which includes 88 distinct Luminex bead-conjugated non-HLA antigens (**Supplementary Figure 1**). Assays were conducted according to the manufacturer’s protocol. Briefly, 40 µL of antigen-coated bead mix and 10 µL of patient serum were added to each well of a pre-wetted filter plate and incubated for 30 minutes at room temperature in the dark with rotation. After washing (1 × 100 µL, then 2 × 250 µL wash buffer), 50 µL of PE-conjugated anti-human IgG (1:10 dilution) was added and incubated for another 30 minutes. Plates were washed and beads resuspended for acquisition on a Luminex 100 system (Luminex Corp., Austin, TX). Antibody binding was reported as the median fluorescence intensity (MFI) of IgG binding on the Luminex 100 (Luminex).

Each run included internal positive and negative control sera and two built-in control beads to ensure assay validity. Reference ranges were established using sera from 100 non-transfused male donors. The 15 most-reactive samples were excluded, and for each antigen, a mean MFI was calculated from the remaining 85. A test sample was considered positive if its MFI was ≥3× the mean. The non-HLA panel reactive antibody (PRA) percentage was calculated as: (number of positive beads ÷ 88) × 100. Non-HLA antibodies were stratified into quartiles (top 25% assigned to Q4, top 25-50% assigned to Q3, bottom 25-50% assigned to Q2, and bottom 25% assigned to Q1) based on reactivity scores for descriptive analysis. Chord plots were generated based on the number of sera showing positivity for each antibody.

### 2.4 Endothelial cell crossmatch assay

The endothelial cell crossmatch (ECXM) detects recipient IgG antibodies that bind to antigens on human endothelial cells, including non-HLA targets. As endothelial cells form the primary immunologic interface between the graft and recipient, ECXM enables the identification of clinically relevant allo- and autoantibody responses not captured by standard lymphocyte-based crossmatch assays.

To evaluate the association between non-HLA antibodies and ECXM reactivity, sera from the recipients were incubated with six different cultured human endothelial cell lines. To minimize false-positive results due to preformed HLA antibodies, only sera from eight transplant recipients without detectable HLA antibodies against these cell lines were selected. Following incubation, unbound antibodies were removed, and bound IgG was detected using a fluorochrome-labeled anti-human IgG secondary antibody. Antibody binding was quantified by flow cytometry and expressed as median channel shift (MCS) relative to a negative control serum, and 50 MCS was used as the cutoff for positive ECXM.

### 2.5 Allograft biopsies and definition of rejection and graft survival

For recipients with preformed DSA or cPRA ≥80%, protocol biopsies were performed at 6 months post-transplant and repeated at 12 months if inflammation was present. Cause biopsies were obtained when clinically indicated for suspected rejection. Collectively, biopsy data were available for all recipients within one year after transplantation. Rejection was defined according to the 2015 Banff criteria. The AMR included active (C4d^+^ with or without DSA, or C4d^-^/DSA^+^ with MVI ≥2), chronic (C4d^+^ with or without DSA, or C4d^-^/DSA^+^ with MVI ≥2), and probable (C4d^-^/DSA^+^ with MVI = 1) forms. Acute cellular rejection (ACR) encompasses Banff grades 1–3 and chronic active T–cell–mediated rejection.

Outcomes, along with donor and recipient baseline characteristics, were obtained from the Organ Procurement and Transplantation Network (OPTN) database, electronic health records (EHR), and the HLA laboratory database. Death-censored graft survival was determined using OPTN data available through December 31, 2025. Graft survival was defined as freedom from dialysis or re-transplantation.

### 2.6 Statistical analysis

Data analysis was conducted following verification of assay validity using MFI values from internal positive and negative control beads. Background-corrected MFI (BCM) was calculated for each bead using manufacturer-provided raw and background MFI values. Comparisons between two independent groups were performed using unpaired, two-tailed Student’s t-tests. A p-value < 0.05 was considered statistically significant. For analyses involving individual non-HLA antibodies, statistical significance was defined as an adjusted p-value ≤ 0.05. All analyses were performed using GraphPad Prism version 10 (GraphPad Software, San Diego, CA). Primary outcomes were analyzed using Kaplan–Meier survival curves, and log-rank tests were applied to compare graft survival between groups.

## 3 Results

### 3.1 Baseline characteristics of the study cohorts

**Table 1** summarizes the demographic and clinical characteristics of 167 kidney transplant recipients stratified by pfDSA, dnDSA, and graft outcomes. The median age at transplantation was 47 years, with a predominance of female recipients (64%). The cohort was racially and ethnically diverse, comprising approximately one-third Hispanic/Latino recipients, one-fourth African American recipients, 22.2% White recipients, and 18.6% Asian recipients. Body mass index, donor characteristics, dialysis duration, and primary disease etiology were comparable across groups, with most recipients being highly sensitized (cPRA ≥ 80%). Overall, baseline characteristics were well balanced among the study groups.

### 3.2 Non-HLA antibody prevalence in kidney transplant sera

Among pre-transplant samples, the majority (74.8%) had a non-HLA PRA of 1–10%, with 16.2% between 11–20%, and 7.2% ranging from 21–57%. In post-transplant samples, 80.8% had a non-HLA PRA of 1–10%, 9.6% between 11–20%, and 5.4% between 21–39%. No significant associations were observed between non-HLA PRA levels (pre- or post-transplant) and recipient age, ABO blood group, or HLA cPRA levels (Pearson r = 0.025; P = 0.75).

Pairwise correlation analysis identified several strongly associated antibody clusters. ANXA2Rand IYD showed strong correlations with DEXI, SDF1B, VWF, and NPHS1 (r > 0.84). Antibodies against collagen types I, III, IV, V, and VI (excluding type II) were highly correlated with myosin (r > 0.91), while FN1 exhibited an even stronger correlation with myosin (r > 0.95). Vascular endothelial growth factor A (VEGFA) and EDIL3 were also strongly correlated with transferrin (r > 0.88) (**Supplementary Figure 1**).

### 3.3 IgA nephropathy and African ancestry are associated with elevated non-HLA PRA

**Figure 2:**
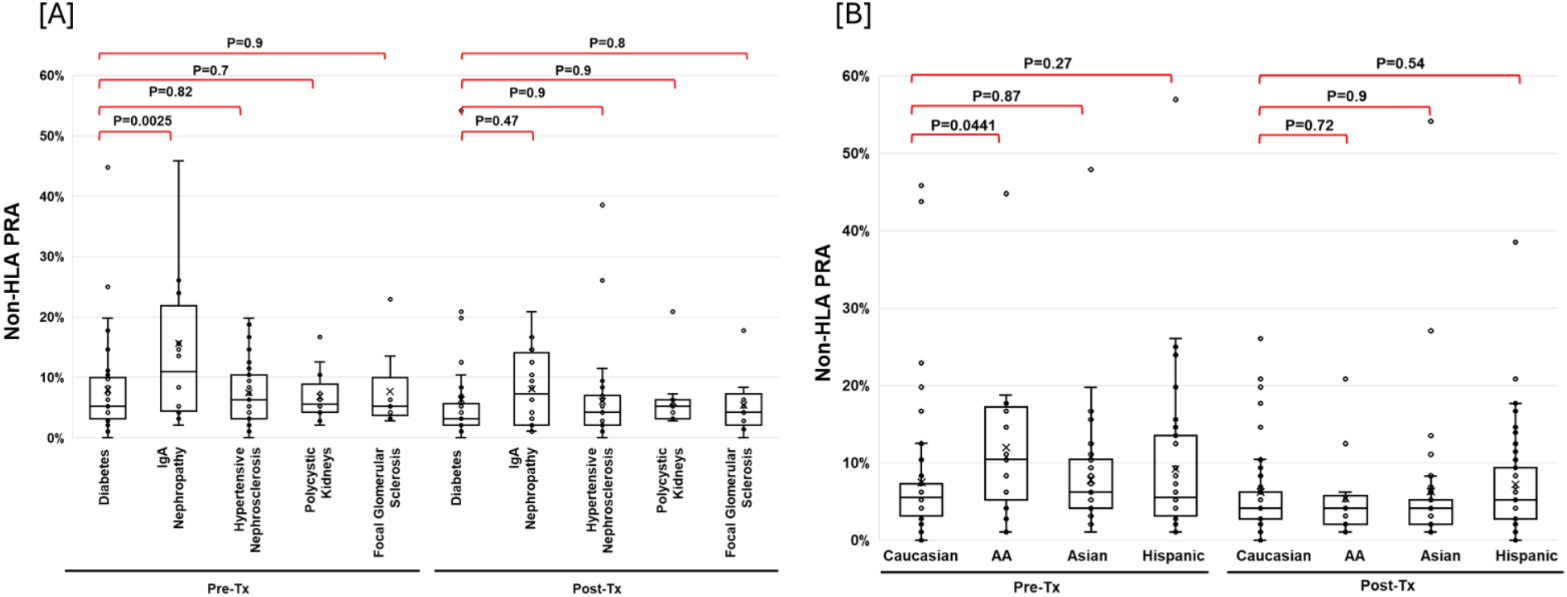
Association of Non-HLA PRA levels with underlying kidney disease and ethnic background. Non-HLA panel reactive antibody (PRA) levels in recipients stratified by end-stage kidney disease (ESKD) etiology **(A)**. Recipients with IgA nephropathy had significantly higher non-HLA PRA levels compared to those with diabetic nephropathy (P = 0.0025). Comparison across racial/ethnic groups **(B)** showed that African American recipients had significantly higher non-HLA PRA levels than Caucasian recipients (P = 0.044).

Pre-transplant non-HLA PRA was significantly higher in patients with IgA nephropathy compared to those with diabetic nephropathy as the cause of end-stage kidney disease (P = 0.0025; **Figure 2**). Additionally, African American recipients exhibited significantly higher non-HLA PRA than Caucasian recipients (P = 0.044; **Figure 2**).

### 3.4 Pregnancy and prior transplants enhance non-HLA antibody formation

**Figure 3:**
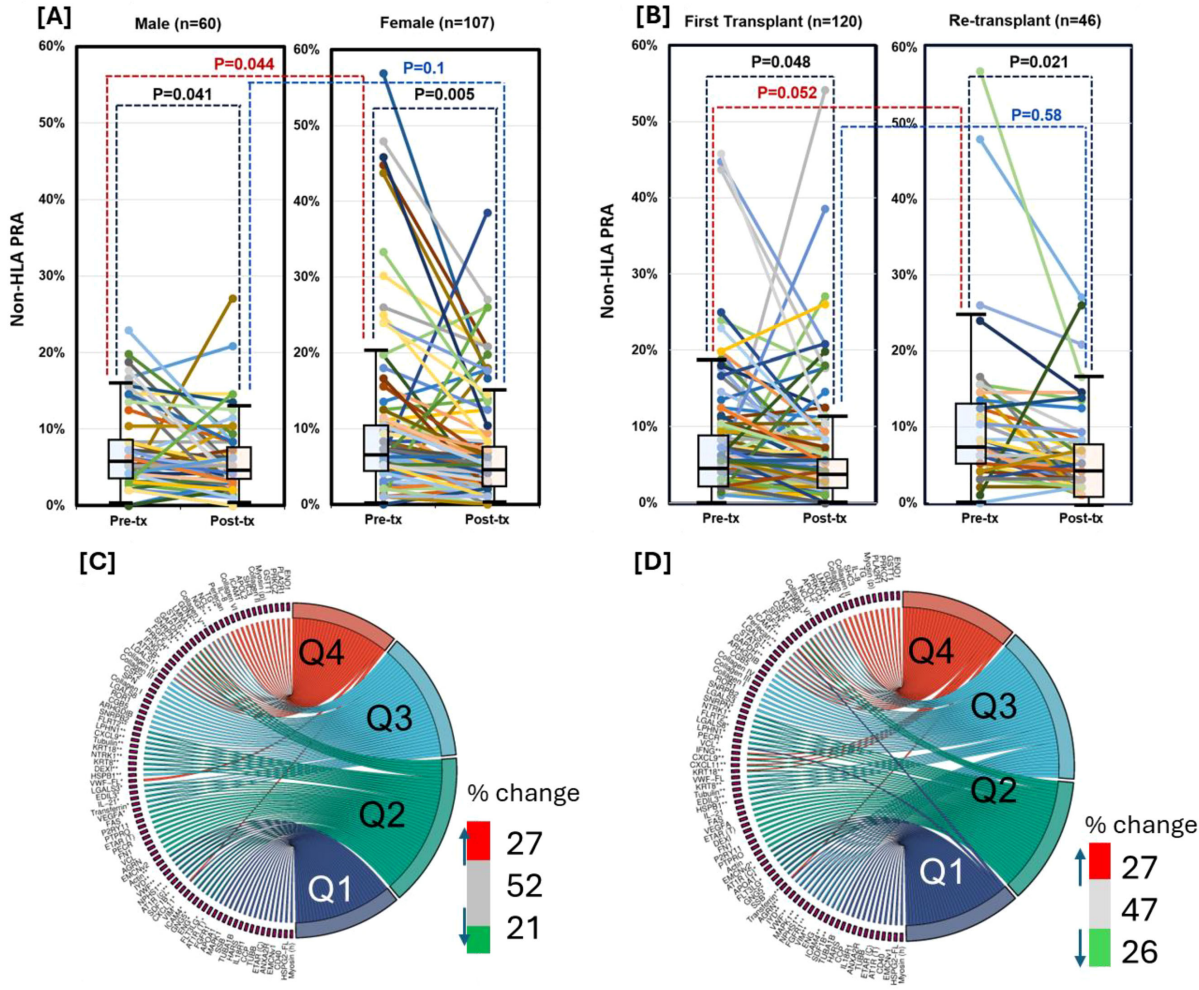
Effect of pregnancy and repeat transplantation on non-HLA antibody burden and specificity. Non-HLA panel reactive antibody (PRA) levels in pre- versus post-transplant (Txp) sera for male (n = 60) and female (n = 107) recipients. Both groups showed a significant post-Txp decline in non-HLA PRA (P = 0.005 for females, P = 0.041 for males) **(A)**. Pre-transplant non-HLA PRA was significantly higher in repeat transplant candidates compared to first transplant candidates (P = 0.052), consistent with allo-sensitization through prior transplant exposure **(B)**. Sex-based interquartile shifts in individual pre-transplant antibody abundance reveal that CXCL11 and VWF-FL reactivity decreased in both sexes (Q4→Q1 and Q4→Q3, respectively), whereas antibodies against LMNA, STAT6, GAPDH, and SNRPN increased in females (Q2→Q4), indicating sex-specific modulation of non-HLA responses **(C)**. Quartile migration of individual pre-transplant antibodies in repeat versus first transplant recipients showed ~30% of non-HLA antibodies shifting up or down in abundance, with notable decreases in FGF2, SPN, and NGF, and a marked increase in transferrin (Q1→Q4), indicating both qualitative and quantitative reshaping of the antibody profile with repeat transplantation **(D)**.

Exposure to alloantigens through pregnancy or transplantation has been associated with increased HLA antibody formation (23–25). To investigate whether similar sensitization influences non-HLA antibody production, we analyzed pre-transplant non-HLA PRA in relation to sex and transplant history. Pre-transplant non-HLA PRA levels were significantly higher in females compared to males (P = 0.044; **Figure 3**), suggesting that pregnancy-associated alloantigen exposure may induce non-HLA antibody production. Likewise, re-transplant candidates exhibited significantly elevated non-HLA PRA compared to first-time transplant candidates (P = 0.052; **Figure 3**), indicating that prior transplant exposure also contributes to non-HLA sensitization.

Quartile-based antibody reactivity analysis revealed that antibodies to CXCL11 and VWF-FL decreased in females compared to males, shifting from Q4 to Q1 and Q4 to Q3, respectively (**Figure 3**). In contrast, antibodies against LMNA, STAT6, GAPDH, and SNRPN were more abundant in females, with reactivity shifting from Q2 to Q4. Among re-transplant candidates, approximately 30% of non-HLA antibodies exhibited interquartile shifts—both increases and decreases—relative to first-time transplant candidates (**Figure 3**). Notably, antibodies against FGF2, SPN, and NGF showed decreased abundance, while antibodies against transferrin increased markedly, shifting from Q1 to Q4.

### 3.5 Temporal dynamics of non-HLA PRA distinguish kidney transplant outcomes

**Figure 4:**
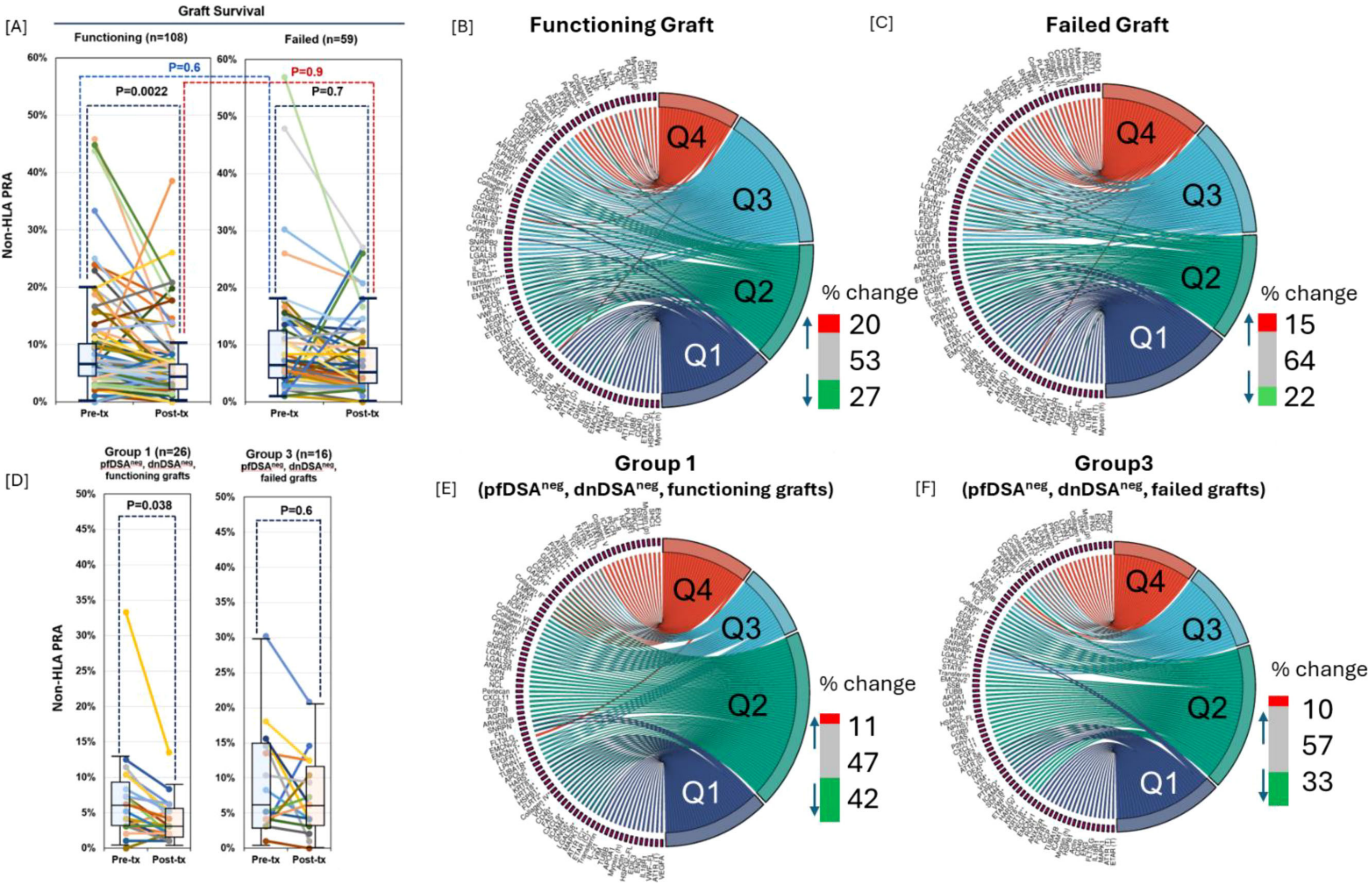
Longitudinal changes in non-HLA Ab burden and abundance distribution post-transplantation in recipients with functioning and failed grafts. Non-HLA panel reactive antibody (non-HLA PRA) levels pre- and post-transplant (Txp) among kidney transplant recipients with functioning grafts (n = 108) and graft failure (n = 59). There was no significant difference in non-HLA PRA between groups at either time point; however, only recipients with functioning grafts showed a significant decrease in non-HLA PRA from pre- to post-Txp (P = 0.0022) **(A)**. Interquartile shifts in antibody abundance for 60 non-HLA Abs between pre- and post-Txp samples among recipients with **(B)** functioning and **(C)** failed grafts. Quartiles (Q1–Q4) were defined as described in methods. Among functioning grafts, 20% of antibodies shifted to higher quartiles, 27% shifted to lower quartiles, and 53% remained unchanged; in failed grafts, 15% increased, 22% decreased, and 64% remained unchanged **(B, C)**. In recipients without preformed or *de novo* HLA-DSA, post-Txp non-HLA PRA significantly declined in those with functioning grafts (Group 1, left panel; P = 0.038) but not in those with graft failure (Group 3, right panel) **(D)**. Interquartile movements in Group 1 **(E)** and Group 3 **(F)** show more pronounced antibody contraction among Group 1 recipients (42% shifted to lower quartiles) compared to Group 3 (33%). Notable antigen-specific changes included, SSB shifting from Q1 to Q4 in both graft outcome groups; APOL2 decreasing in both groups; EMCNv2 decreasing from Q4 to Q2 in Group 3; and VEGFA and ATP5B decreasing from Q4 to Q2 in Group 1 **(E, F)**.

There was no statistically significant difference in non-HLA PRA levels between recipients with graft failure and those with functioning grafts in either pre-transplant or post-transplant serum samples (**Figure 4**). However, within the functioning graft group, non-HLA PRA significantly decreased post-transplant compared to pre-transplant (P = 0.0022), whereas levels remained unchanged in the graft failure group (**Figure 4**).

To evaluate the dynamics of specific antibody changes, we stratified all 60 non-HLA antibodies into quartiles based on abundance (Q4 = highest, Q1 = lowest; see Methods). We then tracked interquartile shifts from pre- to post-transplant: Among recipients with functioning grafts, 20% of antibodies shifted to higher quartiles, 27% to lower quartiles, and 53% remained unchanged (**Figure 4**). In recipients with graft failure, 15% shifted to higher quartiles, 22% to lower quartiles, and 64% showed no change (**Figure 4**).

**Figure 5:**
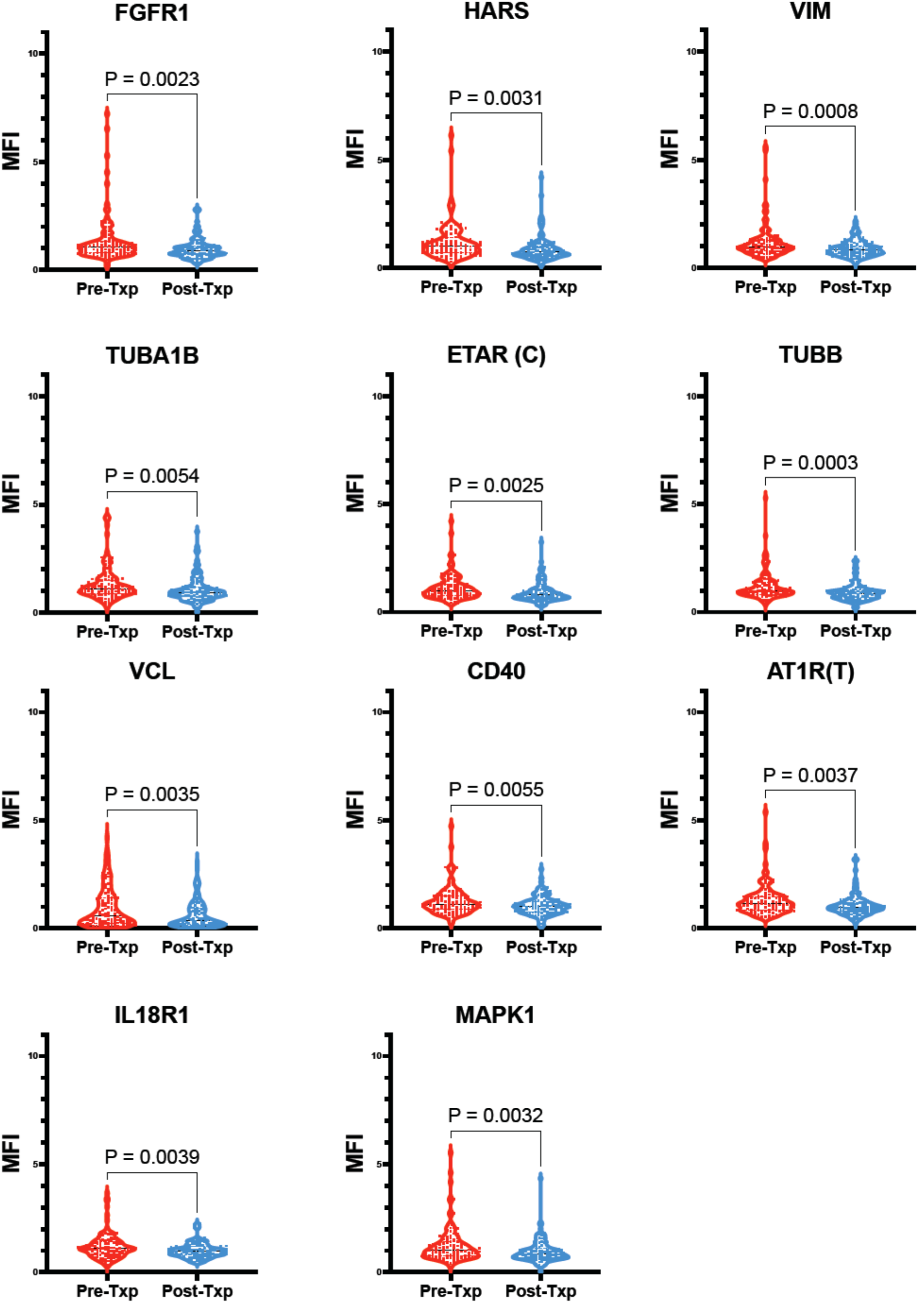
Non-HLA Abs with significant decrease in post-Txp sera compared to pre-Txp sera among patients with functioning grafts. 11 non-HLA-Abs [Fibroblast Growth Factor Receptor 1 (FGFR1), Histidyl-tRNA Synthetase (HARS), Vimentin (VIM), Tubulin Alpha 1B (TUBA1B), Tubulin Beta Class I (TUBB), Vinculin (VCL), Cluster of Differentiation 40 (CD40), Interleukin 18 Receptor 1 (IL18R1), Mitogen-Activated Protein Kinase 1 (MAPK1), Angiotensin II Type 1 Receptor (AT1R, T), and Endothelin Receptor Type A (ETAR, C)] were significantly decreased in post-Txp compared to pre-Txp among patients with functioning grafts.

When all non-HLA Abs were assessed for their changes pre-Txp to post-Txp sera among the patients with functioning grafts, the following antibodies were decreased significantly: Fibroblast Growth Factor Receptor 1 (FGFR1), Histidyl-tRNA Synthetase (HARS), Vimentin (VIM), Tubulin Alpha 1B (TUBA1B), Endothelin Receptor Type A (ETAR, C), Tubulin Beta Class I (TUBB), Vinculin (VCL), Cluster of Differentiation 40 (CD40), Angiotensin II Type 1 Receptor (AT1R, T), Interleukin 18 Receptor 1 (IL18R1), and Mitogen-Activated Protein Kinase 1 (MAPK1) were significantly decreased in post-Txp sera (**Figure 5**).

Subgroup analysis of recipients without pre-transplant DSA and *de novo* DSA revealed no significant change in non-HLA PRA from pre- to post-transplant in those with graft failure (Group 3; **Figure 4**, right panel). In contrast, recipients with functioning grafts (Group 1) showed a significant post-transplant reduction in non-HLA PRA (P = 0.038; **Figure 4**, left panel). In Group 1 (functioning graft), 11% of antibodies increased, 42% decreased, and 47% remained in the same quartile (**Figure 4**). In Group 3 (graft failure), 10% increased, 33% decreased, and 57% were unchanged (**Figure 4**). Specifically, anti-EMCNv2 shifted from Q4 to Q2 in Group 3, while anti-VEGFA and anti-ATP5B shifted from Q4 to Q2 in Group 1.

### 3.6 Evaluation of non-HLA PRA dynamics post-transplantation

**Figure 6:**
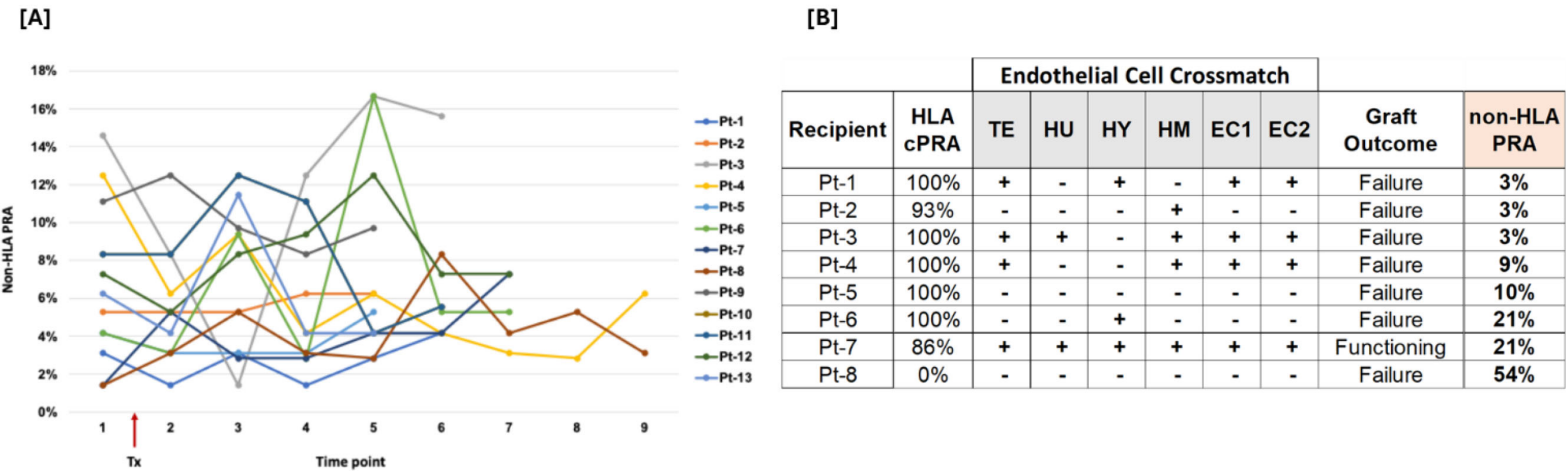
Temporal changes of non-HLA Abs and their association with endothelial cell crossmatch. Longitudinal tracking of non-HLA PRA in 13 kidney transplant recipients over 5–9 months post-transplantation revealed considerable variability and lack of a consistent trajectory. Some patients (e.g., Pt-1) showed an initial post-transplant drop followed by a rebound, while others (e.g., Pt-2) demonstrated sustained decline. Conversely, cases like Pt-7 and Pt-8 exhibited a rise in non-HLA PRA immediately post-transplant compared to baseline **(A)**. Endothelial cell crossmatch (ECXM) results obtained using six different endothelial cell lines (TE - TeloHAEC, HU - HUVEC, HY - EA.hy926, and HM - HMEC-1, EC1 and EC2) and sera from eight transplant recipients without detectable HLA antibodies against these cell lines showed no association with graft outcomes, non-HLA PRA levels or ECXM reactivity **(B)**.

To assess temporal changes in non-HLA PRA following kidney transplantation, 13 recipients were monitored longitudinally over 5–9 months with multiple post-transplant time points. The analysis revealed that non-HLA PRA levels were not stable and did not follow a uniform trajectory. For example, Pt-1 and Pt-2 exhibited elevated pre-transplant non-HLA PRA, followed by a marked decline at 2 months post-transplant. Thereafter, Pt-1 showed a rebound in non-HLA PRA levels, whereas Pt-2 exhibited a sustained decline through months 3–9. In contrast, Pt-7 and Pt-8 showed an increase in non-HLA PRA immediately post-transplant compared to baseline (**Figure 6**).

### 3.7 No correlation between non-HLA antibodies, ECXM reactivity, and kidney graft outcome

Crossmatching was performed using six different endothelial cell lines. Serum samples from eight kidney transplant recipients without detectable HLA antibodies against these cell lines were selected to evaluate the association between non-HLA PRA, ECXM status, and graft failure. ECXM results showed no correlation with graft outcomes or non-HLA PRA levels (**Figure 6**).

### 3.8 Kidney transplant outcomes

The overall incidence of ACR was 6% (n = 10), while AMR occurred in 16.2% (n = 27) of recipients within the first-year post-transplant (**Table 2**). Nearly all patients who experienced rejection—specifically, all with AMR and all but one with ACR—developed dnDSA. The highest rates of AMR were observed in recipients with both pfDSA and dnDSA (Groups 6 and 8), occurring in 41.7% and 42.9% of patients, respectively. In contrast, no rejection episodes were observed among DSA-negative recipients, regardless of graft function status (Groups 1 and 3).

**Table 2:** Distribution of acute cellular rejection (ACR) and antibody-mediated rejection (AMR) according to preformed and *de novo* donor-specific antibody (DSA) status and graft survival within one year post-transplant.

	Preformed DSA	*de novo* DSA	Graft survival	ACR	AMR
				% (n)	% (n)
All Patients (N=167)				6 (10)	16.2 (27)
Group-1 (N=26)	Negative	Negative	Functioning	0	0
Group-2 (N=28)	Negative	Positive	Functioning	3.6 (1)	28.6 (8)
Group-7 (N=26)	Positive	Negative	Functioning	3.8 (1)	0
Group-8 (N=28)	Positive	Positive	Functioning	3.6 (1)	42.9 (12)
Group-3 (N=16)	Negative	Negative	Failed	0	0
Group-4 (N=28)	Negative	Positive	Failed	17.9 (5)	7.1 (2)
Group-5 (N= 3)	Positive	Negative	Failed	0	0
Group-6 (N=12)	Positive	Positive	Failed	16.7 (2)	41.7 (5)

**Figure 7:**
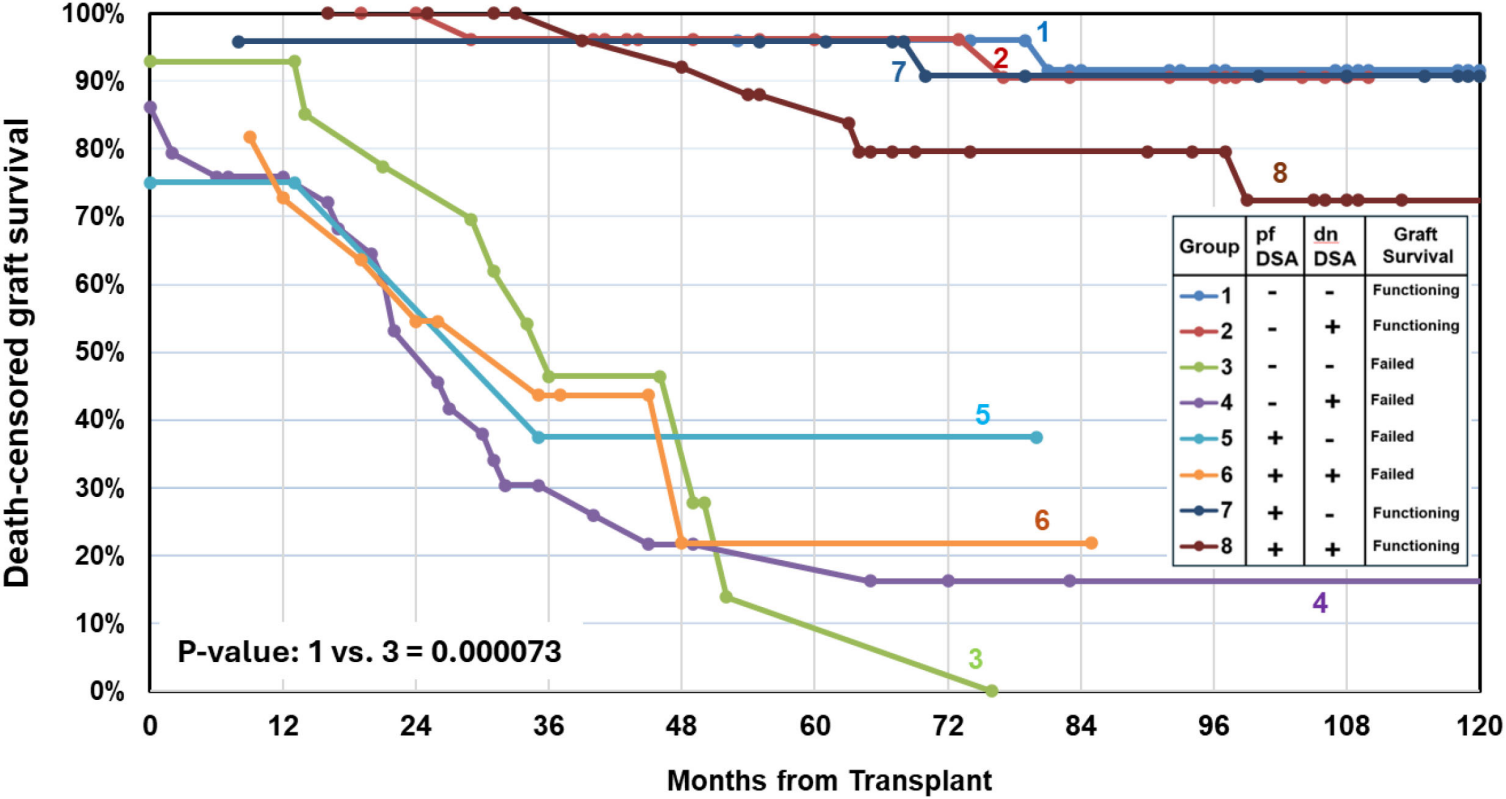
Death-censored graft survival by DSA status. Kaplan–Meier curves depict death-censored graft survival across eight groups defined by preformed (pf) and *de novo* (dn) donor-specific antibody (DSA) status. Overall, the presence of pfDSA or dnDSA did not consistently predict graft survival. Notably, comparison between Groups 1 and 3, both negative for pfDSA and dnDSA, revealed markedly different survival outcomes (p = 0.000073), suggesting that factors beyond HLA-specific humoral responses—such as variations in non-HLA PRA trajectories—may contribute to graft loss.

**Figure 7** illustrates death-censored graft survival across eight study groups stratified by pfDSA and dnDSA status. Overall, the presence of pfDSA or dnDSA did not uniformly predict graft survival. Interestingly, Groups 1 and 3, both negative for pfDSA and dnDSA, exhibited markedly different outcomes, suggesting that additional factors—such as distinct non-HLA PRA trajectories—may contribute to graft loss. Similarly, Groups 6 and 8, both positive for pfDSA and dnDSA, showed divergent graft survival patterns accompanied by differing non-HLA PRA trajectories.

## 4 Discussion

A longitudinal analysis of 167 kidney transplant recipients, profiled using an 88-antigen non-HLA array, revealed that non-HLA humoral sensitization is common in the transplant population and likely reflects cumulative immune exposures—such as infections, inflammation, autoimmunity, and exposure to allogeneic tissues (26). While global non-HLA PRA declined significantly post-transplant (from 9% to 6%, P < 0.001), this reduction was observed exclusively in recipients with functioning grafts. In contrast, patients with graft failure, including those without pre-formed or *de novo* donor-specific HLA antibodies (DSA), exhibited no significant change, indicating that non-HLA alloimmunity can contribute to graft injury independently of the HLA system (27). These findings reinforce the concept of non-HLA antibodies as a distinct immunologic axis in transplantation (28, 29). Antigen-specific analyses further demonstrated a net decline in reactivity in stable grafts (27% decreased vs. 20% increased), suggestive of immune modulation or accommodation in the setting of successful graft function.

The post-transplant decline in multiple non-HLA antibody specificities likely reflects immunologic modulation associated with graft stability. Several of the affected targets are involved in fundamental cellular and immune processes. FGFR1 and MAPK1 are key mediators of growth factor–dependent signaling, while HARS plays a role in protein synthesis. Cytoskeletal and adhesion-related proteins—including VIM, TUBA1B, TUBB, and VCL—are essential for maintaining cell integrity and facilitating tissue remodeling and have been implicated in injury and repair responses. Immune-associated targets, such as CD40 and IL-18R1, participate in costimulatory and pro-inflammatory signaling, suggesting the attenuation of immune activation in recipients with stable grafts. Additionally, reduced reactivity to AT1R and ETAR—G-protein–coupled receptors involved in vascular regulation and increasingly recognized non-HLA targets in transplantation—may indicate diminished endothelial or vascular immune stress in this cohort. Notably, high-titer responses to select targets, such as SSB and collagens V and VI, persisted across all outcome groups. Together, these data highlight the dynamic, graft-dependent behavior of non-HLA antibodies and underscore their relevance for immunologic monitoring and risk stratification beyond conventional HLA metrics.

All patients in this cohort received the same conditioning and maintenance therapy; therefore, the decline in non-HLA antibody levels observed in recipients with functioning grafts is unlikely to be attributable to immunosuppression. Although mechanistic data are lacking, it is plausible that stable graft function fosters a tolerogenic environment that facilitates the contraction of non-HLA humoral responses, whereas graft failure may sustain their persistence.

### 4.1 IgA nephropathy and African ancestry are associated with elevated non-HLA PRA

Non−HLA PRA was higher in IgA−nephropathy recipients than in people with diabetes (P < 0.003). IgA nephropathy is characterized by immune complex deposition and systemic immune activation, which may promote the development of non-HLA antibodies targeting autoantigens, endothelial, or minor histocompatibility antigens (30). In contrast, diabetic nephropathy arises from metabolic and hemodynamic stress due to hyperglycemia and lacks a significant adaptive immune component, resulting in a lower likelihood of non-HLA sensitization (31).

Non−HLA PRA was higher in African American than Caucasian recipients (P < 0.05). African American individuals exhibit greater genetic diversity, including HLA and non-HLA polymorphisms, which may enhance alloimmune responsiveness. Elevated baseline immune activation and inflammatory cytokine levels further contribute to increased humoral reactivity. Additionally, higher rates of clinical sensitization (e.g., transfusions, infections) and potential environmental or epigenetic influences may predispose this population to heightened non-HLA antibody formation (32).

### 4.2 Non-HLA PRA and HLA cPRA represent distinct alloimmune pathways in kidney transplantation

Notably, the extent of non-HLA antibody reactivity, as measured by non-HLA PRA scores, was independent of conventional sensitization metrics; specifically, pre-transplant non-HLA PRA did not correlate with calculated HLA-PRA (cPRA). Non-HLA PRA and cPRA reflect immunologically distinct processes. While cPRA measures antibodies directed against polymorphic HLA molecules, non-HLA PRA captures reactivity to non-HLA targets such as endothelial cell antigens (e.g., AT1R, ETAR) and autoantigens. These antibodies arise via different sensitization pathways—HLA antibodies typically result from transfusions, pregnancies, or transplants, whereas non-HLA antibodies may emerge from tissue injury, inflammation, autoimmunity, or infections. Mechanistically, HLA antibody formation is driven by adaptive immune responses, whereas non-HLA antibodies may involve innate immunity, loss of tolerance, or non-classical antigen presentation (33). Consequently, the lack of correlation between non-HLA PRA and cPRA highlights their independent origins and supports the need for comprehensive alloimmune risk profiling in transplant recipients.

### 4.3 Pregnancy and re-transplantation shape the breadth and specificity of non-HLA antibody responses

Alloantigen exposure from pregnancy and prior transplantation enhances non-HLA antibody production, resulting in elevated pre-transplant non-HLA PRA levels in females and re-transplant recipients. Pregnancy exposes the maternal immune system to paternal HLA and non-HLA antigens, while transplantation introduces a diverse set of donor-derived antigens, including endothelial and intracellular targets. Sex-specific differences in non-HLA antibody profiles were evident, with females exhibiting decreased reactivity to CXCL11 and VWF-FL, but increased levels of antibodies against LMNA, STAT6, GAPDH, and SNRPN. These patterns suggest that pregnancy-associated sensitization influences both the breadth and specificity of the non-HLA humoral response. Re-transplantation was associated with dynamic shifts in non-HLA antibody repertoires, with approximately 30% of antigens showing interquartile changes. While some antibodies (e.g., FGF2, SPN, NGF) declined, likely due to regulatory mechanisms, others, such as anti-transferrin antibodies, increased markedly, indicating antigen-specific amplification rather than generalized immune activation.

### 4.4 Temporal instability of non-HLA PRA post-transplantation

Serial monitoring of 13 kidney transplant recipients over 5–9 months revealed substantial temporal variability in non-HLA PRA levels, characterized by patient-specific fluctuations rather than consistent trends. Patterns included initial declines with subsequent rebounds, sustained decreases, and early post-transplant increases, reflecting dynamic immune processes influenced by prior sensitization, graft-related injury, and the effectiveness of immunosuppression. These observations highlight the non-linear, individualized nature of non-HLA antibody responses, driven by diverse antigen targets and variable immune regulation. The findings caution against reliance on single post-transplant time points for immune risk assessment and emphasize the importance of longitudinal monitoring. Overall, non-HLA PRA represents a distinct and adaptable immunologic axis, with important implications for transplant surveillance and outcome prediction.

### 4.5 Antigen−specific insights and network biology

We aimed to assess the titer of non-HLA Abs and investigated whether the abundance correlated with the antigen’s specific subcellular location or antigenicity. In terms of the subcellular location of the antigens, highly abundant Abs exhibited diverse subcellular locations reflecting their varied biological functions. ENO1, GAPDH, GSTT1, PRKCZ, PRKCH, SHC3, and Myosin are predominantly cytoplasmic, with some also associated with the plasma membrane (34, 35). Sutherland et al. reported that kidney transplant patients with elevated levels of antibodies against protein kinase C zeta (PKCζ; gene PRKCZ) developed rejection, that they are likely to end up with a graft loss (36). We detected the highest median titers against cytosolic enzymes (ENO1, GAPDH, GSTT1), matrix proteins (collagens I–VI, fibronectin), and vascular mediators (VEGFA). Post−transplant, antibodies to ETAR, FGFR1, MAPK1, and vinculin declined significantly (adjusted P < 0.05), paralleling reductions in nephrin and AT1R. Alpha-enolase (ENO1) is an autoantigen associated with cancer and autoimmune diseases, where anti-ENO1 antibodies have been detected in patient sera (37). Correlation mapping confirmed three robust clusters: (i) ANXA2R/IYD with VWF and NPHS1, (ii) collagens with myosin and fibronectin, and (iii) VEGFA/EDIL3 with transferrin. These networks imply coordinated targeting of structurally contiguous graft compartments, supporting mechanistic hypotheses of endothelial activation and epitope spreading, phenomena well described in chronic active AMR.

### 4.6 Integration with contemporary literature and clinical relevance

Our findings corroborate and extend the rapidly growing evidence base on non-HLA immunity in solid-organ transplantation. The STAR 2022 workgroup review concluded that more than 90% of kidney candidates harbor detectable non-HLA antibodies, yet standardized thresholds, reproducible platforms, and prospective validation remain major unmet needs. In the largest single−center cohort to date (n = 934), Senev et al. demonstrated that every 10 additional positive non−HLA specificities increased the hazard of developing AMR histology by 14 % (HR = 1.14; 95 % CI 1.03–1.27) and that this relationship persisted in the absence of donor−specific HLA antibodies (29). Our trajectory−based data refine these observations by showing that the direction and magnitude of change—rather than the static burden—that best discriminates functional outcomes.

Evidence for antigen−specific pathogenicity remains mixed. In pediatric recipients, Comoli et al. found that high-level anti-GSTT1 antibodies (4th-quartile MFI) were independently associated with AMR and persisted throughout follow-up (18), whereas an adult Spanish cohort reported no effect of anti-GSTT1 on graft filtration or survival (38). Such discordance highlights the heterogeneity of non-HLA targets and suggests the probable influence of age, ethnicity, immunosuppression, and assay methodology. Beyond GSTT1, multicenter analyses have linked antibody breadth against intracellular signaling proteins (STAT6), cytoplasmic enzymes (ENO1), and membrane receptors (AT1R, ETAR) to graft dysfunction; however, causality remains to be proven (10, 11). Cardiac-transplant data echo these kidney findings: Butler et al. reported that non-HLA antibody load synergizes with low-level HLA-DSA to accelerate transplant vasculopathy, while See et al. observed phenotypic convergence between non-HLA-mediated and mixed-antibody vasculopathy, suggesting convergent mechanisms of microvascular injury (10, 11). Together with our observation that non−HLA PRA declines only in functioning grafts, these studies support a paradigm in which dynamic monitoring of non−HLA immunity offers additive prognostic value beyond baseline serostatus. The STAR workgroup emphasized several research priorities—cross-platform standardization, antigen discovery, longitudinal biorepositories, and synergy modeling with HLA antibodies (28)—all of which align with the prospective, multi-center agenda we outline below.

In this cohort, rejection events—including both ACR and AMR—were absent in DSA-negative recipients (Groups 1 and 3), regardless of graft function status. These findings indicate that, in the absence of HLA-directed humoral immunity, classic forms of rejection detectable on biopsy are unlikely to occur. Instead, non-HLA antibodies may contribute to graft dysfunction through alternative mechanisms such as antibody-dependent cellular cytotoxicity (ADCC), endothelial activation, or other non-classical effector pathways.

Despite the well-established association between DSA and graft injury, our data demonstrate that neither the presence of pfDSA nor dnDSA uniformly predicted graft survival. This heterogeneity underscores the multifactorial nature of allograft injury, where non-HLA antibodies and innate immune activation may modulate outcomes even in DSA-negative settings. The observation that Groups 1 and 3—both lacking pfDSA and dnDSA—exhibited significantly different graft outcomes supports the growing recognition that complement-independent mechanisms, microvascular inflammation, and innate immune activation may contribute to graft loss in select recipients. Similarly, the divergent survival trajectories among DSA-positive recipients (Groups 6 and 8) suggest that the immunologic milieu—including the evolution of non-HLA PRA trajectories—may influence the clinical impact of DSA.

### 4.7 Strengths and limitations

This study’s non-randomized, retrospective design reflects real-world transplant heterogeneity and enables the investigation of antibody dynamics in clinically relevant subgroups. It represents the largest longitudinal non-HLA antibody dataset, featuring paired serum sampling and high-resolution antigen-level tracking. Key strengths include its temporal design and the ability to monitor dynamic changes in non-HLA reactivity over time. However, several limitations should be acknowledged. First, the antigen panel lacked coverage of certain clinically relevant targets (e.g., GSTT1, LIMS1, ARHGDIB). Second, the non-randomized, single-center design introduces potential confounding from unmeasured clinical and immunological variables, such as prior sensitization (including blood transfusion), donor characteristics, infection, inflammation, and variations in immunosuppressive therapy. Third, sampling bias may be present, as the longitudinal subset (n = 13) was selected solely based on sample availability rather than clinical outcome, which could affect the interpretation of antibody kinetics. Additionally, antibody reactivity was measured solely by MFI using the Luminex platform, which quantifies binding strength but does not assess functional or pathogenic potential, underscored by the observed lack of correlation between non-HLA PRA and ECXM results. Finally, as a single-center study—with relatively small sample sizes in certain subgroups—generalizability is limited, and the findings should be considered hypothesis-generating, warranting validation in larger, prospective, multicenter studies with standardized sampling and outcome assessment.

### 4.8 Conclusion

Non-HLA antibodies are highly prevalent and dynamically regulated following kidney transplantation, providing clinically relevant insights that extend beyond conventional HLA-based assessments. However, their clinical implementation remains limited by biological heterogeneity, uncertain pathogenic relevance, and technical variability across detection platforms. Although their contribution to DSA-negative rejection is increasingly recognized, the absence of standardized assays and validated biomarkers continues to impede routine clinical use.

Advancing the clinical utility of non-HLA antibody testing will require comprehensive functional characterization, assay harmonization, and rigorous prospective validation. Although the non-HLA antibody data presented in this manuscript do not directly establish a strong association with graft outcomes, our findings support the establishment of a prospective, multicenter initiative integrating broad antigen discovery approaches (e.g., PhIP-Seq, spatial transcriptomics) with serial immune monitoring and protocol biopsies in a larger cohort of study participants to clarify causality and therapeutic responsiveness. Collaborative standardization efforts, such as those led by the STAR consortium, will be critical to enabling reliable inter-laboratory comparisons. Ultimately, integrating both HLA and non-HLA immune profiles will be essential to achieving truly personalized and durable outcomes in kidney transplantation.

## Data Availability

The raw data supporting the conclusions of this article will be made available by the authors, without undue reservation.

## Supplementary material

Supplementary Figure 1 Network analysis of co-varying non-HLA antibody specificities. Correlation heatmaps and cluster analysis identified strong positive co-variation among specific non-HLA antibodies. Notable clusters included: ANXA2R–IYD–DEXI, collagen types I–VI with myosin, and VEGFA–EDIL3 with transferrin (r > 0.84–0.95). These clusters suggest coordinated immune recognition of structurally or functionally related antigen sets in kidney transplant recipients.
