# Supplementary material for: Non-HLA antibody trajectories may predict kidney transplant outcomes
Source: Front Immunol. 2026 Feb 24;17:1729129. doi: 10.3389/fimmu.2026.1729129 (PMC12971436; doi:10.3389/fimmu.2026.1729129)
Supplement: Supplementary Figure 1 — Network analysis of co-varying non-HLA antibody specificities. Correlation heatmaps and cluster analysis identified strong positive co-variation among specific non-HLA antibodies. Notable clusters included: ANXA2R–IYD–DEXI, collagen types I–VI with myosin, and VEGFA–EDIL3 with transferrin (r > 0.84–0.95). These clusters suggest coordinated immune recognition of structurally or functionally related antigen sets in kidney transplant recipients. [file Image1.pdf]

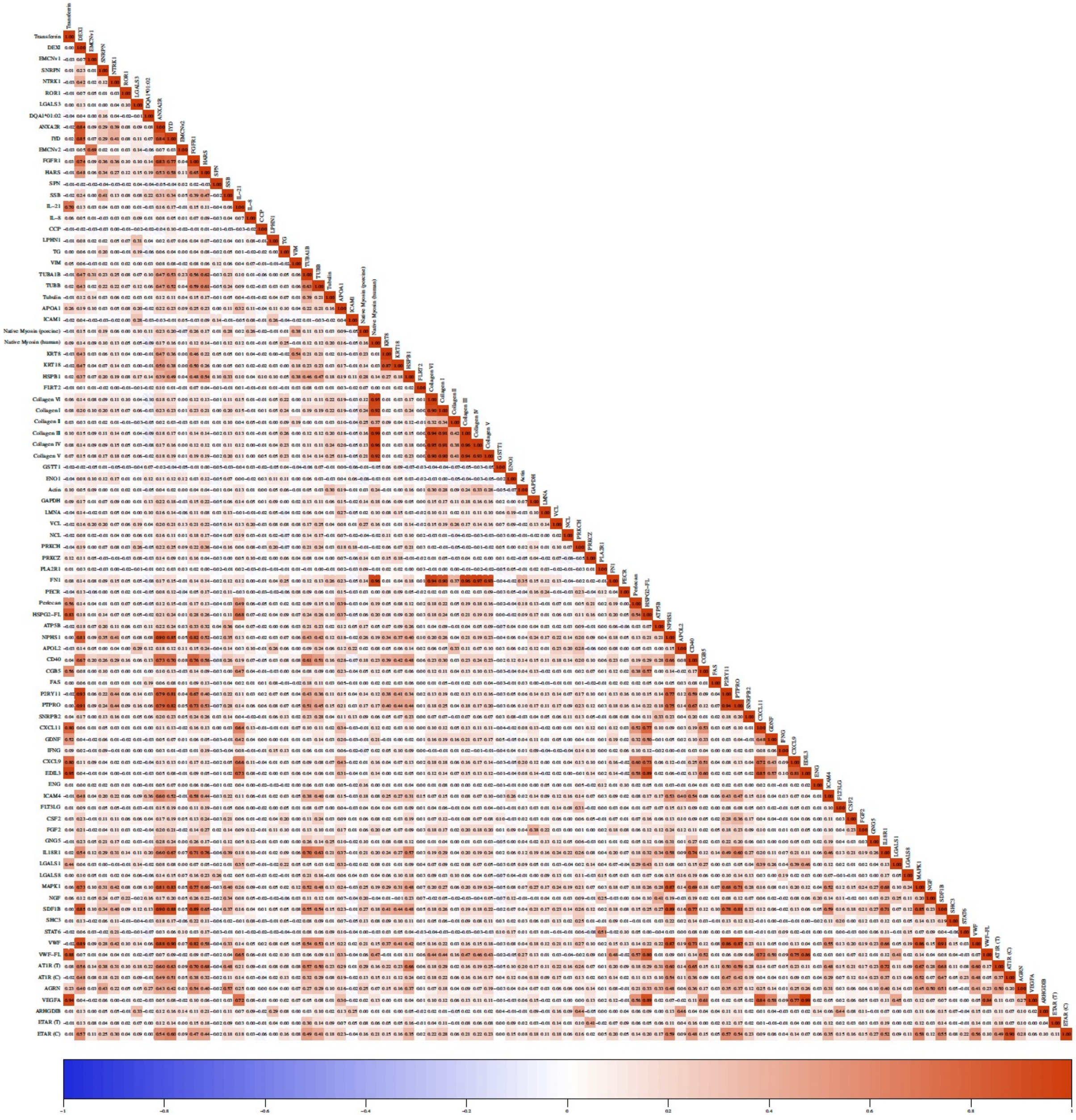
Transferrin
DEXI
EMCNv1
SNRPN
NTRK1
ROR1
LGALS3
DQA1*01:02
ANXA2R
IYD
EMCNv2
FGFR1
HARS
SPN
SSB
IL−21
IL−8
CCP
LPHN1
TG
VIM
TUBA1B
TUBB
Tubulin
APOA1
ICAM1
Native Myosin (porcine)
Native Myosin (human)
KRT8
KRT18
HSPB1
FLRT2
Collagen VI
Collagen I
Collagen II
Collagen III
Collagen IV
Collagen V
GSTT1
ENO1
Actin
GAPDH
LMNA
VCL
NCL
PRKCH
PRKCZ
PLA2R1
FN1
PECR
Perlecan
HSPG2−FL
ATP5B
NPHS1
APOL2
CD40
CGB5
FAS
P2RY11
PTPRO
SNRPB2
CXCL11
GDNF
IFNG
CXCL9
EDIL3
ENG
ICAM4
FLT3LG
CSF2
FGF2
GNG5
IL18R1
LGALS1
LGALS8
MAPK1
NGF
SDF1B
SHC3
STAT6
VWF
VWF−FL
AT1R (T)
AT1R (C)
AGRN
VEGFA
ARHGDIB
ETAR (T)
ETAR (C)
-1
-0.8
-0.6
-0.4
-0.2
0
0.2
0.4
0.6
0.8
1
